# New Candidate Genes for a Chicken Pectoralis Muscle Weight QTL Identified by a Hypothesis-Free Integrative Genetic Approach

**DOI:** 10.3390/genes17010062

**Published:** 2026-01-05

**Authors:** Akihiro Furuta, Akira Ishikawa

**Affiliations:** Laboratory of Animal Genetics and Breeding, Graduate School of Bioagricultural Sciences, Nagoya University, Nagoya 464-8601, Japan

**Keywords:** correlation analysis, *CDH17*, gene expression, quantitative trait loci, *RNF151*

## Abstract

**Background/Objectives**: Identifying candidate genes underlying quantitative trait loci (QTL) in poultry has traditionally required labor-intensive positional cloning. Previous studies using an F_2_ population derived from native Japanese Nagoya (NAG) and White Plymouth Rock (WPR) breeds revealed a major QTL on chromosome 2 affecting 3-week body weight and 4-week pectoralis muscle weight. This study aimed to identify candidate genes for this QTL using a hypothesis-free integrative genetic approach. **Methods**: We employed a multi-step analytical framework combining QTL remapping, transcriptome analysis, gene enrichment analysis, haplotype frequency comparison, and correlation analysis. QTL remapping was performed using individual traits and their first principal component (PC1) in 239 F_2_ chickens. RNA-sequencing (RNA-seq) of liver tissue was conducted for F_2_ individuals with extreme PC1 scores, followed by reverse transcription quantitative polymerase chain reaction (RT-qPCR) validation. **Results**: QTL remapping refined the 95% confidence interval to a chromosome 2 region containing 329 genes. RNA-seq analysis identified 23 differentially expressed genes (DEGs) within this interval. Although gene enrichment analysis initially highlighted GATA binding protein 6 (*GATA6*) as a potential candidate, RT-qPCR in NAG, WPR, and F_1_ chickens showed no significant expression differences, excluding *GATA6*. Haplotype frequency and correlation analyses prioritized cadherin-17 (*CDH17*) as the strongest candidate gene and ring finger protein 151 (*RNF151*) as a secondary candidate. **Conclusions**: Our hypothesis-free integrative approach effectively refined candidate genes for a chromosome 2 QTL influencing early growth and pectoralis muscle weight. *CDH17* and *RNF151* represent promising targets for functional validation and may support marker-assisted selection to improve muscle-related traits in chickens.

## 1. Introduction

Most phenotypic traits of agricultural, medical, and biological importance in animals, including poultry, livestock, model organisms, and humans, are governed by complex genetic architectures and are influenced by environmental factors. Quantitative trait loci (QTL) analyses and genome-wide association studies (GWASs) have identified numerous genomic regions associated with these traits across nearly all chromosomes. However, pinpointing the causal genes or specific genetic variants underlying these QTLs remains challenging. This difficulty arises because most QTLs have small phenotypic effects, lie within relatively broad confidence intervals or regions of high linkage disequilibrium, and often reside in non-coding regions of the genome [1,2,3,4,5]. Identification of causal genes and genetic variants responsible for quantitative traits is essential to elucidating their complex genetic architecture. Particularly, for economically important traits in poultry and livestock, such knowledge can be directly applied to gene-assisted selection programs to improve breeds with desirable characteristics tailored to specific production goals.

In recent decades, it has become increasingly clear that a single analytical method is insufficient to prioritize potential candidate genes from the hundreds located in a given QTL confidence interval [1,4]. Consequently, various integrative strategies have been developed, which combine multiple lines of evidence to narrow down the list to a limited number of plausible causal genes [6,7]. In chickens, a representative example is the integrative genetic approach developed by our previous study [8]. This approach combines QTL remapping, RNA-sequencing (RNA-seq) analysis, reverse transcription quantitative PCR analysis (RT-qPCR), haplotype frequency analysis, and correlation analysis. It employs the original QTL mapping population derived from a cross between two breeds with distinct phenotypes and does not rely on any a priori assumptions. Using this approach, Ochiai et al. [8] successfully narrowed down 333 genes within the confidence interval of a QTL on chromosome 4 associated with innate fear behavior to just two strong candidate genes, based on two chicken groups exhibiting extreme behavioral phenotypes selected from an F_2_ population derived from a cross between the native Japanese Nagoya (NAG) breed, characterized by a timid temperament, and the White Leghorn breed, which exhibits a normal control temperament. In the present study, we apply this integrative approach to prioritize candidate genes in our chicken population.

More than 45 native Japanese breeds have been established in various regions of Japan since around the Heian Period (794–1185 Common Era) [9]. Although these breeds were primarily developed for ornamental purposes, crowing, and behavioral traits, they are also renowned for their excellent meat and egg quality, making them valuable but untapped genetic resources for the poultry industry. In recent decades, commercial Jidori chickens, certified under the Japanese Agricultural Standards (JAS), have been produced by crossbreeding selected native Japanese breeds with Western chicken breeds. These Jidori chickens are highly prized for their flavorful, high-quality meat, which far surpasses that of common broilers. Among native Japanese breeds, the NAG chicken is unique in having been established solely for meat and egg production, and it is well known for its exceptional meat and egg quality [9].

Several QTL studies using crossbreds between Japanese and Western chicken breeds have revealed important aspects of the genetic architectures underlying body weight and growth [10,11,12], meat yield and quality [13,14], egg quality [15,16,17], and temperament [18,19]. Essa et al. [12] and Ishikawa et al. [14] performed QTL analyses of body weight at 3 weeks of age and three pectoralis muscle weight traits (pectoralis minor, pectoralis major, and total pectoralis muscle weight) at 4 weeks of age in an F_2_ population obtained from a cross between the native Japanese NAG breed and the high-growth White Plymouth Rock (WPR) breed. They identified QTLs with main effects on body weight and pectoralis muscle weight on chromosomes 2 and 4. At all loci detected, the WPR-derived allele exhibited dominant or additive effects relative to the NAG-derived allele. Notably, the peaks of logarithms of the odds (LOD) scores for the chromosome 2 QTL affecting body weight and the three pectoralis muscle weight traits were located very close, with pectoralis minor weight showing the highest LOD score (6.0) and accounting for 11.1% of the phenotypic variance.

In this study, we aimed to identify candidate genes underlying the chromosome 2 QTL associated with variation in body weight at 3 weeks of age and pectoralis muscle weight at 4 weeks of age between the NAG and WPR breeds. To accomplish this, we employed the hypothesis-free integrative genetic approach developed by Ochiai et al. [8] to prioritize candidate genes within the QTL region.

## 2. Materials and Methods

### 2.1. Animals and Traits

An F_2_ cross population of 239 birds (119 males and 120 females) was previously obtained by crossing between NAG and WPR breeds [14,20]. Body weight was measured weekly from hatching to 4 weeks of age [14,20]. At 4 weeks of age, in our previous study [20], blood and organ/tissue samples (pectoral minor and major muscles, liver, white adipose tissue, and gizzard) were collected from healthy birds, including 239 F_2_ individuals, 29 WPR individuals, 16 NAG individuals, and eight F_1_ individuals at Nagoya University. All samples were stored at −80 °C until analysis. Weights of the pectoralis minor and major muscles were measured at 4 weeks of age, and their sum was referred to as total pectoralis muscle weight [14]. Of these traits, those used in this study were body weight at 3 weeks of age, pectoralis major muscle weight, pectoralis minor muscle weight, and total pectoralis muscle weight. These traits were previously reported to be significantly affected by the chromosome 2 QTL at the genome-wide 5% level [12,14], and trait data for the NAG and WPR breeds and their F_1_ and F_2_ chickens are presented in Supplementary Table S1. The effects of environmental factors (dam, sex, and hatch date) on raw trait data were assessed, and those found to be significant at the nominal 5% level were statistically removed (Supplementary Table S2). Adjusted trait values were standardized to zero mean ± 1 standard deviation and used in subsequent analyses of this study. All laboratory procedures and handling of the chickens were performed in accordance with protocols established by the Nagoya University Animal Research Committee (approval numbers: 2016022303, 2017030205, A230001, A240037).

### 2.2. Principal Component Analysis

Principal component analysis (PCA) was conducted on body weight, pectoralis major muscle weight, pectoralis minor muscle weight, and total pectoralis muscle weight using a correlation matrix of JMP Pro software version 17.2.0 (SAS Institute Japan Ltd., Tokyo, Japan). A total of 239 F_2_ individuals were ranked based on their first principal component (PC1) scores, and individuals with the highest and lowest PC1 scores were selected for subsequent RNA-seq and RT-qPCR analyses.

### 2.3. QTL Remapping

A genetic linkage map was constructed using the Kosambi map function based on 313 single nucleotide polymorphism (SNP) markers previously developed by restriction site-associated DNA sequencing (RAD-seq) analysis [12]. To refine the QTL position on chromosome 2, a single-QTL genome scan was performed on the four trait values and PC1 scores of 239 F_2_ individuals using the Haley-Knott regression of the R/qtl package version 4.3.0 [21]. To find a sex-specific QTL, single-QTL genome scans were performed on males and females separately, followed by QTL-by-sex interaction analysis. Genome-wide 5% and 10% significance thresholds were obtained through 10,000 permutation tests of R/qtl. The 95% confidence interval of the detected QTL was estimated based on the 1.8 drop method of LOD scores; the percentage of phenotypic variance explained by the QTL, and the additive and dominance effects of the QTL were estimated by R/qtl.

### 2.4. RNA-Seq Analysis

Total RNA was extracted from the livers of three F_2_ individuals of each sex exhibiting the highest and lowest PC1 scores. The extraction was performed using TRI reagent (Cosmo Bio Co., Ltd., Tokyo, Japan), phase separation with chloroform, and RNA precipitation with isopropanol (both from FUJIFILM Wako Pure Chemical Co., Tokyo, Japan). RNA concentrations were measured using a NanoDrop One spectrophotometer (Thermo Fisher Scientific, Tokyo, Japan). RNA-seq analysis and subsequent sequence data analysis were outsourced to Rhelixa, Inc. (Tokyo, Japan). Briefly, RNA libraries were prepared using the NEBNext^®^ UltraTMII Directional RNA Library Prep Kit for Illumina (New England Biolabs Japan Inc., Tokyo, Japan) and sequenced with 150 bp paired-end reads on an Illumina NovaSeq 6000 platform. Raw read quality was assessed using FastQC version 0.11.7 (https://www.bioinformatics.babraham.ac.uk/projects/fastqc (accessed on 24 April 2019)). Adapter sequences and low-quality reads (<20 bp) were removed using Trimmomatic version 0.38 with the following parameters: ILLUMINACLIP:path/to/adapter.fa:2:30:10 LEADING:20 TRAILING:20 SLIDINGWINDOW:4:15MINLEN:36 [22]. Trimmed reads were aligned to the chicken reference genome RefSeq GRCg6a (https://www.ncbi.nlm.nih.gov/grc/chicken/data (accessed on 21 February 2022)) using HISAT2 version 2.1.0 [23]. Raw read counts were normalized using the Relative Log Expression method, and differential gene expression analysis was performed with DESeq2 version 1.24.0 [24]. Differentially expressed genes (DEGs) were identified at a nominal threshold of *p* < 0.05, rather than using Benjamini-Hochberg adjusted *p*-values, to avoid excluding potentially relevant genes.

The RNA-seq data were deposited in the DDBJ Sequence Read Archive under accession numbers DRR540127-DRR540132 (aishikawa-0018_Run_0001-0006) for males and DRR540133-DRR540138 (aishikawa-0019_Run_0001-0006) for females. All data can be searched using these accession numbers on the DDBJ Sequence Read Archive (DRA) website (https://www.ddbj.nig.ac.jp/dra/index.html).

### 2.5. Functional Gene Enrichment and Pathway Analyses

Functional gene enrichment and pathway analyses of the DEGs identified by RNA-seq were performed using Metascape [25], a web-based platform for a comprehensive gene/protein list annotation and enrichment analysis based on Gene Ontology (GO) and Kyoto Encyclopedia of Genes and Genomes (KEGG) pathways. Metascape automatically converted the input gene identifiers to human Entrez Gene ID using the EggNOG and HomoloGene databases.

### 2.6. RT-qPCR Analysis

Total RNA was extracted from the livers of eight individuals each from NAG and WPR breeds, eight F_1_ individuals, 20 F_2_ individuals with the highest PC1 scores, and 18 F_2_ individuals with the lowest PC1 scores. Before RT-qPCR, 1.0 µg of total RNA was reverse-transcribed into cDNA using the PrimerScript RT reagent Kit with gDNA Eraser (Takara Bio, Otsu, Japan). RT-qPCR analysis was conducted using the StepOnePlus Real-Time PCR system (Thermo Fisher Scientific, Tokyo, Japan) and TB Green Premix Ex Taq II (Tli RNaseH Plus) (Takara Bio, Otsu, Japan). To select suitable internal control genes among five candidates (tyrosine 3-monooxygenase/tryptophan 5-monooxygenase activation protein zeta (*YWHAZ*), TATA-box binding protein (TBP), actin, beta (*ACTB*)*,* ribosomal protein L32 (*RPL32*), and glyceraldehyde-3-phosphate dehydrogenase (*GAPDH*)), amplification efficiencies of target and control genes were evaluated using a quantitative relative standard curve with four serial dilutions (10, 2, 0.4, and 0.08 ng/µL). Pairs of target and control genes with similar amplification efficiencies were analyzed using the 2^−ΔΔCt^ method. All reactions were conducted in triplicate. Sequences of the primer pairs used are shown in Table 1. Gene expression levels were compared among NAG, WPR, and F_1_ groups using one-way analysis of variance (ANOVA), followed by Tukey’s honest significant difference (HSD) post hoc test in JMP Pro software.

### 2.7. Haplotype Frequency Analysis

Diplotypes for two extreme F_2_ groups with the highest and lowest PC1 scores (10–25 individuals in each group) were determined based on genotypes at five SNP marker loci located within the 95% confidence interval of the QTL. F_2_ individuals carrying recombinant haplotypes were excluded from the analysis. Haplotype frequencies were compared between the two extreme F_2_ groups using Pearson’s chi-square test.

### 2.8. Correlation Analyses

Gene expression levels were compared between 20 F_2_ individuals with the highest and 18 individuals with the lowest PC1 scores. To determine whether the genes were candidates for the QTL, correlation analyses were performed between gene expression levels and each of the pectoralis muscle weights, body weight, and PC1 scores in the two extreme F_2_ groups. These analyses were conducted using linear regression models in JMP Pro software.

## 3. Results

### 3.1. Principal Component Analysis

To precisely map the QTL on chromosome 2, PCA was performed using the 239 F_2_ chickens previously used for the initial QTL analysis [12,14]. This analysis incorporated four traits: body weight, pectoralis major muscle weight, pectoralis minor muscle weight, and total pectoralis muscle weight. PCA reduced these four traits to two principal components (PC1 and PC2), which explained 86.9% and 11.4% of the total variance, respectively (Supplementary Figure S1 and Table 2). All four traits exhibited positive factor loadings on PC1. Among them, the three muscle traits showed higher loadings (0.97–0.98) than body weight (0.71), indicating that PC1 primarily reflects variation in pectoralis muscle weight. Thus, F_2_ chickens with higher PC1 scores tended to have heavier pectoral muscles. In contrast, PC2 showed a high positive factor loading for body weight (0.61) and negative loadings for the three muscle traits (−0.17 to −0.16), suggesting that PC2 represents a trade-off between early body weight and muscle development. (Table 2). Furthermore, PCA was performed using only the three pectoralis muscle weights. PC1 alone explained 97.8% of the total variance, with all three traits exhibiting strong positive factor loadings (0.99) (Supplementary Figure S1 and Table 2). Based on these results, two types of PC1 scores summarizing variation in all four traits and in the three muscle traits were used as composite traits in the subsequent QTL analysis.

### 3.2. QTL Remapping

A linkage map comprising 313 SNP markers was constructed using 239 F_2_ chickens and is shown in Supplementary Figure S2 and Supplementary Table S3. Based on this linkage map (expressed in centiMorgan, cM), QTL analysis was performed for body weight, three muscle weights, and two types of PC1 scores obtained using all four traits and the three muscle traits. Supplementary Table S4 summarizes the genome-wide significance LOD thresholds used in single-QTL genome scans for sex-combined and sex-specific data across all traits.

As shown in Table 3 and Supplementary Figure S3, single-QTL genome scans using sex-combined data revealed that the LOD peaks for all traits exceeded the genome-wide 5% significance thresholds. The LOD peaks of the three muscle traits and the two types of PC1 scores were located at nearly the same map position (111.4–114.8 Mb) on chicken chromosome 2. In contrast, the LOD peak for body weight was detected at a slightly different location (104.7 Mb). For all traits, alleles derived from the WPR breed increased trait values. Among these traits, pectoralis minor muscle weight showed the highest LOD score (6.08), accounting for 11.18% of the trait variance. In contrast, pectoralis major muscle weight exhibited the lowest LOD score (3.66), explaining 6.89% of the variance (Table 3). The 95% confidence intervals for the three muscle weights ranged from 100.8 Mb to 125.3 Mb, overlapping with that for body weight (98.0–127.4 Mb). To avoid overlooking potential candidate genes, the widest interval, spanning 29.4 Mb from 98.0 to 127.4 Mb, was defined as the QTL region in this study and used for subsequent candidate gene identification.

Single-QTL genome scans using sex-specific data revealed different LOD score patterns between males and females (Supplementary Figure S3). In males, all five traits except for pectoralis major muscle weight exceeded at least the genome-wide 10% significance threshold, whereas none of the traits exceeded this threshold in females. Nevertheless, sex-by-QTL interaction effects for all traits did not reach the genome-wide 10% threshold, suggesting the absence of sex-specific QTLs for any of the traits.

### 3.3. RNA-Seq Analysis

RNA-seq analysis was performed on two extreme F_2_ groups of each sex with the highest and lowest PC1 scores. This analysis identified a total of 896 DEGs across the entire genome at nominal *p* < 0.05 in either sex. Within the QTL region (98.0 to 127.4 Mb) on chromosome 2, 329 genes were annotated, of which 23 were identified as DEGs. Among them, three genes (ENSGALG00000050994, ENSGALG00000052336, and impact RWD domain protein (*IMPACT*)) were excluded for further analysis because they showed zero expression in three or more of the six individuals of each sex examined. Consequently, 20 DEGs within the QTL region were retained for further analysis, as listed in Table 4.

### 3.4. Functional Gene Enrichment and Pathway Analyses

To identify functional candidate genes in the QTL region based on known gene functions, enrichment analysis was performed using Metascape [25]. This analysis revealed significant enrichment in six GO terms involving seven DEGs (Supplementary Table S5). Among these DEGs, GATA binding protein 6 *(GATA6*) was associated with four key GO terms: growth, developmental process, cellular process, and response to stimulus. Furthermore, *GATA6* was linked to a total of 12 biological terms (Table 5), the highest number among all DEGs analyzed. Cadherin-17 (*CDH17*) was linked to three terms related to immune system processes, whereas ring finger protein 151 (*RNF151*) showed no associations (Table 5 and Supplementary Table S5). These findings suggested that *GATA6* is the most plausible functional candidate gene in the QTL region.

### 3.5. RT-qPCR Analysis

Except for one gene (ENSGALG00000053658), for which suitable primers could not be designed (Table 4), RT-qPCR analysis was performed on 19 genes using liver samples from the parental NAG and WPR breeds and their F_1_ offspring, to further narrow down the list of candidate genes. Among these, seven genes showed significant differences in expression among the three breeds (*p* < 0.05, two-way ANOVA), and two additional genes exhibited marginal differences (*p* = 0.052–0.060) (Table 6). Notably, *GATA6*, previously identified as the most plausible functional candidate gene based on gene enrichment analysis, did not show a significant expression difference (*p* = 0.25) and was therefore excluded from the list of potential candidates. Consequently, the remaining nine genes were subjected to haplotype frequency analysis.

### 3.6. Haplotype Frequency Analysis

Haplotypes of the nine genes that passed the above RT-qPCR screening were inferred based on genotypes at the five SNP marker loci used in the QTL analysis. After excluding individuals with recombinant haplotypes, haplotype frequencies were compared between two extreme F_2_ groups, each comprising sex-combined individuals with the highest and lowest PC1 scores. As shown in Table 7, only two genes, *CDH17* and *RNF151*, exhibited marginally significant differences in haplotype frequencies (*p* = 0.08, Pearson’s chi-square test), suggesting a potential association with the QTL. To avoid prematurely excluding possible candidates, these two genes were retained for subsequent screening.

### 3.7. Association Analyses

Liver expression levels of the *CDH17* and *RNF151* genes were compared between two extreme F_2_ groups in each sex, comprising the 10 individuals with the highest and the nine individuals with the lowest PC1 scores. Two-way ANOVA revealed no significant expression differences between the two groups (Supplementary Table S6). However, a highly significant group-by-sex interaction was detected for both genes (*p* = 0.00020–0.00080). Notably, although both genes showed significant expression differences between the parental NAG and WPR breeds, no breed-by-sex interaction was observed (Table 6).

**Figure 1:**
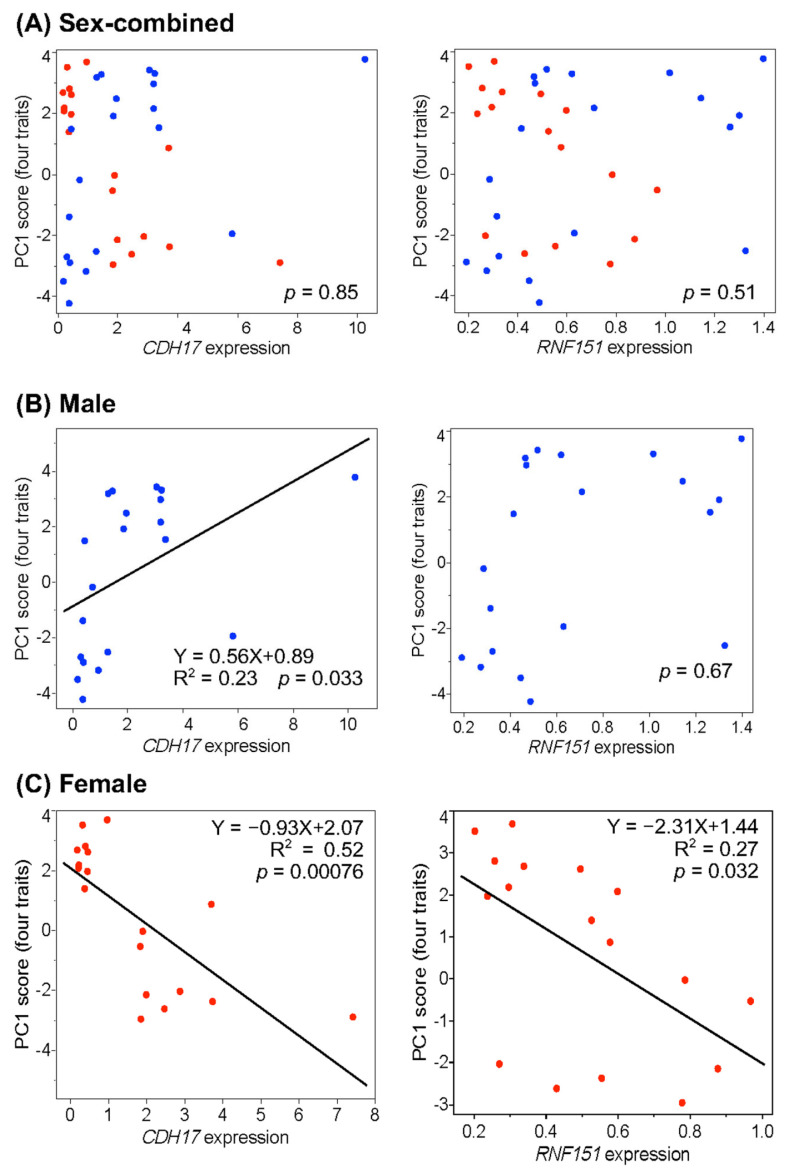
Correlation analysis between liver expression levels of the *CDH17* and *RNF151* genes and the first principal component (PC1) scores for four traits: body weight, pectoralis minor muscle weight, pectoralis major muscle weight, and total pectoralis muscle weight. The analysis was performed by sex using F_2_ individuals with the highest (n = 10) and lowest (n = 9) PC1 scores in each sex. Closed blue and red circles indicate males and females, respectively. (**A**) sex-combined data; (**B**) male data only; (**C**) female data only.

Correlation analyses were conducted between the expression levels of the two genes and all six traits listed in Table 3, using the 20 F_2_ individuals with the highest and the 18 with the lowest PC1 scores. The results are shown in Figure 1 and Supplementary Figure S4. In the sex-combined data, neither gene showed a significant correlation with the PC1 scores for four traits: body weight, pectoralis minor muscle weight, pectoralis major muscle weight, and total pectoralis muscle weight (Figure 1A). In contrast, *CDH17* expression showed a significant positive correlation with PC1 scores in males but a significant negative correlation in females (Figure 1B,C). *RNF151* expression was not significantly correlated with PC1 scores in males but showed a significant negative correlation in females. *CDH17* expression explained 23% of the trait variance (R^2^ = 0.23) in males and 52% in females, while *RNF151* expression accounted for 27% of the variance in females (Figure 1B,C). Similar sex-specific patterns were observed in the correlations between gene expression and the remaining five traits (Supplementary Figure S4). In males, *CDH17* expression explained 20–28% of the variance in corresponding traits, while in females, it accounted for 47–52%. Female *RNF151* expression explained 27–29% of the variance across traits.

## 4. Discussion

In this study, we applied a previously proposed integrative genetic approach using an original segregating F_2_ QTL mapping population in chickens, without relying on prior hypotheses or creating new congenic lines [8]. This strategy enabled us to narrow down the 329 genes located in the QTL region on chromosome 2 to two strong candidate genes: *CDH17* and *RNF151*. Among them, *CDH17* emerged as the most promising candidate, supported consistently across all prioritization steps, whereas *RNF151* was considered a secondary candidate due to weaker evidence in some analytical steps. These findings underscore the effectiveness and efficiency of our integrative approach for prioritizing candidate genes in QTL regions.

Our QTL analysis was performed for four traits: body weight at 3 weeks of age and the weights of three pectoralis muscles at 4 weeks of age. These traits had previously been shown to be significantly affected by the QTL on chicken chromosome 2 [12,14]. In addition to individual traits, we used two types of PC1 scores: one summarizing phenotypic variation across all four traits and another across only the three muscle weight traits, to enhance the power of QTL detection. The re-estimated LOD peak positions and 95% confidence intervals were highly consistent with those reported by Essa et al. [12] and Ishikawa et al. [14], confirming the robustness of the QTL signal. Notably, the LOD peak for body weight was approximately 10 Mb apart from that of the muscle weight traits. However, overlapping confidence intervals and similar sex-specific correlation patterns between liver expression levels of *CDH17*/*RNF151* and the phenotypes suggest that a single genetic locus may exert pleiotropic effects on both body weight and pectoralis muscle weight. Definitive resolution of whether this pattern reflects the pleiotropy of a single locus or the close linkage of two distinct loci will require identification of the causal gene underlying the QTL effect.

Due to the unavailability of pectoralis muscle tissue in this study, we performed transcriptomic analysis using liver tissue to identify candidate QTL genes. The liver is a central metabolic organ involved in various physiological processes, including glucose homeostasis, lipid metabolism, and amino acid turnover [29]. It also plays a key role in the somatotropic axis by producing insulin-like growth factor 1 (*IGF-1*), a hormone crucial for body growth and skeletal muscle development in chickens and other animals [30,31]. In addition, recent studies demonstrated the importance of locally expressed genes, transcripts, and noncoding RNAs in skeletal muscle for muscle growth and development [31,32]. *CDH17* and *RNF151* are not predominantly expressed in skeletal muscle. *CDH17* is mainly expressed in intestinal epithelial cells, although its expression in pectoralis muscle has been reported in chickens [32]. While *RNF151* expression in chickens remains unknown, transcriptomic databases show low but detectable expression of both genes in skeletal muscle of mice and humans (Mouse Genome Informatics v6.24, accessed July 2025; GTEx Analysis Release v10, dbGaP Accession phs000424.v10.p2). Therefore, further transcriptome analysis using pectoralis muscle tissue from our chickens will be essential to clarify the muscle-specific relevance of *CDH17* and *RNF151*.

Functional gene enrichment analysis is a widely used method to identify genes and proteins involved in specific biological processes and diseases. For example, it has been used to discover genes associated with Alzheimer’s disease [33] and cancer [34]. In our study, *GATA6* was initially prioritized as a candidate gene because it is strongly enriched in multiple growth and developmental pathways. However, RT-qPCR analysis revealed no significant expression difference between the parental breeds, and thus, *GATA6* was excluded as a candidate gene. This case illustrates a limitation of enrichment-based approaches, which rely heavily on complete and accurate gene annotation. As noted by Hwang et al. [35], incomplete annotation may lead to false negatives, missing biologically relevant genes. In our study, *CDH17* and *RNF151* were not strongly enriched in annotated biological processes, yet their expression levels were significantly correlated with the phenotypes. This discrepancy highlights the importance of combining enrichment analysis with transcriptomic and phenotypic data when prioritizing candidate genes.

Functionally, *CDH17* has been extensively studied in human gastric cancer, where it is overexpressed and associated with poor prognosis and tumor progression [36,37]. *CDH17* promotes tumor growth through the Ras/Raf/MEK/ERK and β-catenin/GSK-3β signaling pathways [38]. Moreover, its expression has been correlated with fatty liver development in obese rats [39]. While the role of *CDH17* in muscle growth remains unclear, its broad involvement in cell proliferation and tissue remodeling suggests unrecognized roles in muscle development.

*RNF151* is an E3 ubiquitin ligase containing a RING finger domain and is thought to be involved in protein degradation [40]. It is predominantly expressed in the testes of mice and humans, where it regulates spermatogenesis and acrosome formation [41]. Although little is known about *RNF151* in chickens, it has been reported as downregulated during infection with highly pathogenic avian influenza [42]. Our enrichment analysis did not associate *RNF151* with any specific biological processes, leaving its function largely speculative.

Taken together, the roles of *CDH17* and *RNF151* in regulating body weight and pectoralis muscle development remain hypothetical. However, emerging evidence suggests that genes traditionally characterized by specific functions in certain issues can have unexpected roles in other unrelated tissues. A prominent example is myostatin, a myokine best known for regulating muscle mass. Ongaro et al. [43] found that deletion of the myostatin (*Mstn*) gene in mice unexpectedly reduced serum follicle-stimulating hormone (FSH) levels and pituitary expression of the FSHβ subunit (*Fshb*) gene, resulting in reproductive abnormalities in both sexes. These findings revealed a novel muscle-pituitary signaling axis, despite the absence of *Mstn* expression in the mouse pituitary. By analogy, *CDH17* and *RNF151* may also play unexpected roles in muscle growth and development in chickens, beyond their known tissue-specific functions. Functional validation using gene-edited chickens will be essential to determine their causal involvement.

Importantly, the identification of only two candidate genes facilitates downstream functional validation using genome editing tools such as CRISPR/Cas9. To establish causality between a candidate gene and the QTL phenotype, quantitative complementation tests in a uniform genetic background by crossing gene knockout and wild-type lines will be essential, as demonstrated in QTL studies in mice [44,45].

## 5. Conclusions

By applying a prior hypothesis-free integrative genetic approach [8], this study successfully identified *CDH17* as the most promising candidate and *RNF151* as a secondary candidate gene for a QTL affecting body weight and pectoralis muscle weight on chicken chromosome 2. These findings deepen our understanding of the genetic architecture underlying muscle-related traits in chickens and may support the development of more accurate marker-assisted selection strategies in poultry breeding.

## Figures and Tables

**Table 1:** Primer sequences of genes used for RT-qPCR analysis.

Gene ^1^	Forward Primer (5′-3′)	Reverse Primer (5′-3′)	Reference or Accesion No. ^2^
*TBP*	TAGCCCGATGATGCCGTAT	GTTCCCTGTGTCGCTTGC	[26]
*ACTB*	CCAGACATCAGGGTGTGATGG	CTCCATATCATCCCAGTTGGTGA	[27]
*GAPDH*	GAAGGCTGGGGCTCATCTG	CAGTTGGTGGTGCACGATG	[27]
*YWHAZ*	TTGCTGCTGGAGATGACAAG	CTTCTTGATACGCCTGTTG	[28]
*RPL32*	ATGGGAGCAACAAGAAGACG	TTGGAAGACACGTTGTGAGC	[28]
*APCDD1*	AAGTGATGGGCGGAACAGAG	AAGACGTTGAGCAGAGAGGC	NM_001012941.2
ENSGALG00000051993	GGCTTGTTGTTTGCGTTCC	TTGTGATGTGTGTCCTCCTTTGT	ENSGALT00000093901.1
ENSGALG00000054223	CAGTTCCTTTGACCCAGAAAT	ATTGATTTGTGATCGGAGGTC	ENSGALT00000097868.2
ENSGALG00000053416	CGTGGGAGGGCTGTGATTAG	CCAGTGTCTAGCAGCTTGGA	ENSGALT00000099366.1
*TGIF1*	TTTCCTCATCTGCTGGCTCG	GGCGTGCGTTGATAAACCAG	NM_205379.2
*NDC80*	TCTTATGAACTGCCTGACTCAA	CCAAATCAAAGCTGCCACAATC	NM_204477.3
*GATA6*	TGTTTTCACTCTCTCATCGCCTTC	GTACACGCTACCAACACTTGAGAA	NM_001398234.1
*LOC100857837*	GTCTACGCAGCCATCTCAGG	TGTTGAAGTGTTCGCTCAGC	XM_015282646.4
*C2H8ORF22*	CAGCGTTTCTTCCAGCACCT	GCCAACAGTCGGTGGAGATG	NM_001302199.2
*SOX17*	TGTCATTCATGGTCGAGATCCTTG	AAAAACCCGAAGTCTGTACAACGT	NM_001039326.1
*LY96*	TTTCCCCAAAATGAAGCATCC	TGCAGGGTGTCATATTAAGCT	XM_040696637.2
*CA2*	CTGGCTCCCTGACTACTCCA	AGCTCTGACTTCCCTGCTCT	NM_205317.2
*ATP6V0D2*	AGGGTTCAAAGCTGGGATCTT	GCCATAGTCTGTCGTCTGTAGA	NM_001008455.2
*MMP16*	AATGGCAAGAGAGACGTGGA	AGTGTCTCCCCCAATTCCTG	NM_205197.3
*NBN*	AAGCTGTTCAGTCCAGGCAA	ACCAATGGCAGGCTCATCAA	NM_204337.2
*CDH17*	GGGGATGCTGTACGATATTCCTT	GTTCTTGTTCACGATCCAAAGCA	NM_001199495.2
*RNF151*	GCAGAGAGACAGCATCGACA	CCCCTCAGCAGCTCCATTAG	XM_015282944.4
*PTDSS1*	TCCTGGTGTTCGTGCTCTTC	CCCCAGAAATGCCCAAAAGC	NM_001031505.2
*LAPTM4B*	TGCTACCAGATCTTTGACTTTGC	CCAGACACGTAGGGTTCACA	XM_001233338.7

^1^ ENSGALG# indicates the Ensembl Gene ID for *Gallus gallus*; official gene name not yet assigned. ^2^ The sequence data for the genes were retrieved from the NCBI Nucleotide database (https://www.ncbi.nlm.nih.gov/nuccore (accessed on 1 September 2024)).

**Table 2:** Factor loading values obtained by principal component analysis (PCA) of four traits affected by a QTL on chromosome 2.

Trait	All Four Traits		Three Muscle Weights	
	PC1	PC2	PC1	PC2
Body weight	0.79	0.61	NA	NA
Pectoralis minor muscle weight	0.97	−0.16	0.99	0.17
Pectoralis major muscle weight	0.97	−0.17	0.99	−0.15
Total pectoralis muscle weight	0.98	−0.16	0.99	−0.02
Eigenvalue	3.48	0.46	2.93	0.05
% Variance	86.9	11.4	97.8	1.8

PC1, first principal component; PC2, second principal component; NA, not applicable.

**Table 3:** Parameter estimates from single-QTL genome scans using sex-combined data for body weight, three pectoralis muscle weights, and two types of PC1 scores derived from all four traits and from the three muscle traits.

Trait	Pos. ^1^	Nearest Marker	CI ^2^	LOD	% Variance	Additive Effect ^3^	Dominant Effect ^3^
Body weight	86 (104.7)	*SNP79*	45–107 (98.0–127.4)	4.63 **	8.53	0.48 ± 0.10	−0.15 ± 0.18
Pectoralis minor muscle weight	83 (114.8)	*SNP101*	69–99 (101.7–117.0)	6.08 **	11.18	0.54 ± 0.10	−0.26 ± 0.18
Pectoralis major muscle weight	82 (111.4)	*SNP101*	50–101 (100.8–125.3)	3.66 *	6.89	0.41 ± 0.10	−0.26 ± 0.18
Total pectoralis muscle weight	83 (114.8)	*SNP101*	56–100 (101.3–122.7)	4.26 *	7.97	0.45 ± 0.10	−0.26 ± 0.18
PC1 score (all four traits)	83 (114.8)	*SNP101*	62–100 (101.3–120.3)	5.22 **	9.68	0.92 ± 0.19	−0.50 ± 0.33
PC1 score (three muscle traits)	83 (114.8)	*SNP101*	60–100 (101.3–121.0)	4.70 **	8.76	0.81 ± 0.16	−0.45 ± 0.31

^1^ Linkage map position (cM), with the corresponding physical map position (Mb) in parentheses; physical map positions are based on the chicken reference genome GRCg6a. ^2^ 1.8-LOD supports a confidence interval (CI) for the LOD peak, with physical interval (Mb) in parentheses. ^3^ Values are means ± standard error; positive signs for effects in standard deviation units indicate that alleles derived from the WPR breed increased trait values. ** Significant at the genome-wide 1% level; * Significant at the genome-wide 5% level; no asterisk, suggestive significance at the genome-wide 10% level.

**Table 4:** List of 20 differentially expressed genes (DEGs) located within the QTL region on chromosome 2 (98.0 to 127.4 Mb), identified by RNA-seq analysis of two extreme F_2_ groups in each sex, each group comprising three individuals with the highest and lowest PC1 scores.

Gene ^1^	Physical Position (bp) ^2^		Log_2_FC ^3^	
	Start	End	Male	Female
*APCDD1*	98,307,894	98,337,161	**2.97**	**1.08**
ENSGALG00000051993	98,582,916	98,594,266	**−0.84**	−0.03
ENSGALG00000054223	98,750,108	98,758,304	**1.01**	−0.03
ENSGALG00000053416	99,746,306	99,754,086	**4.08**	**−1.47**
*TGIF1*	100,818,249	100,826,684	**1.19**	0.42
*NDC80*	101,172,292	101,191,007	**1.28**	0.56
*GATA6*	102,638,198	102,654,114	−0.08	**−0.70**
*LOC100857837*	107,656,418	107,705,493	**1.62**	0.41
ENSGALG00000053658 ^4^	108,475,567	108,484,328	**1.60**	−0.25
*C2H8ORF22*	108,494,837	108,497,336	**1.72**	**−0.56**
*SOX17*	110,517,253	110,518,918	**2.11**	0.20
*LY96*	118,035,819	118,043,555	−0.18	**0.90**
*CA2*	122,766,240	122,822,370	**−0.70**	**−0.80**
*ATP6V0D2*	122,936,917	122,954,777	**−5.32**	−1.12
*MMP16*	123,570,466	123,745,959	**2.01**	NA
*NBN*	124,273,535	124,294,981	−0.27	**0.64**
*CDH17*	125,879,843	125,906,330	**1.85**	−0.11
*RNF151*	126,031,779	126,041,077	**0.87**	0.06
*PTDSS1*	126,738,656	126,768,617	**−0.73**	**0.62**
*LAPTM4B*	127,225,486	127,287,310	**1.77**	**1.36**

^1^ ENSGALG# indicates the Ensembl Gene ID for *G*.* gallus*; official gene name not yet assigned. ^2^ Positions are based on the chicken reference genome GRCg6a. ^3^ Log_2_ fold change in expression between three individuals with the highest and three with the lowest PC1 scores; bold values indicate statistically significant differences at nominal *p* < 0.05. ^4^ This gene was excluded from the list of candidate genes in subsequent RT-qPCR analysis due to the inability to design suitable primers.

**Table 5:** Genes on chromosome 2 and their associated enriched biological terms identified by Metascape analysis.

Gene	Term	Source Category ^1^	−–Log_10_*p*
*GATA6*	Cell lineage map for neuronal differentiation	WikiPathways	4.13
	Hydronephrosis	GeDiPNet	3.65
	Regulation of growth	GO	3.31
	Chordate embryonic development	GO	3.21
	Embryo development ending in birth or egg hatching	GO	3.15
	Cell population proliferation	GO	3.04
	In utero embryonic development	GO	2.74
	Developmental growth	GO	2.63
	Growth	GO	2.63
	Cellular response to growth factor stimulus	GO	2.48
	Embryonic morphogenesis	GO	2.25
	Response to growth factor	GO	2.40
*CDH17*	Asthma	GeDiPNet	2.34
	Leukocyte activation	GO	2.22
	Cell morphogenesis	GO	2.07
*RNF151*	NS ^2^	NS ^2^	NS ^2^

^1^ Terms were retrieved from the Gene Ontology (GO), WikiPathways, and GeDiPNet databases using Metascape [25]. ^2^ No significantly enriched terms were identified for *RNF151*.

**Table 6:** Differences in the expression levels of 19 genes on chromosome 2 among the WPR breed, the NAG breed, and their F_1_ hybrids for each sex.

Gene ^1^	WPR		F_1_		NAG		*p* Value ^2^		
	M	F	M	F	M	F	B	S	B × S
***APCDD1***	1 ± 0.08	1.03 ± 0.13	0.78 ± 0.04	0.60± 0.09	0.78 ± 0.04	0.60 ± 0.09	0.00080	0.99	0.19
**ENSGALG00000051993**	1 ± 0.04	1.01 ± 0.05	0.88 ± 0.02	0.77 ± 0.10	0.61 ± 0.03	0.57 ± 0.03	0.000013	0.37	0.64
ENSGALG00000054223	1 ± 0.09	1.13 ± 0.19	1.10 ± 0.15	0.76± 0.10	1.11 ± 0.16	0.58 ± 0.06	0.32	0.049	0.082
**ENSGALG00000053416**	1 ± 0.06	1.22 ± 0.07	0.70 ± 0.04	0.84± 0.08	0.87 ± 0.07	1.16 ± 0.14	0.0066	0.013	0.74
*TGIF1*	1 ± 0.13	0.76 ± 0.13	0.78 ± 0.11	0.52 ± 0.08	0.73 ± 0.10	0.82 ± 0.18	0.32	0.27	0.40
***NDC80***	1 ± 0.05	1.32 ± 0.25	0.74 ± 0.08	1.07 ± 0.13	0.72 ± 0.07	1.01 ± 0.04	0.048	0.0060	0.93
*GATA6*	1 ± 0.07	0.79 ± 0.13	1.11 ± 0.17	1.08 ± 0.20	1.19 ± 0.08	1.18 ± 0.19	0.25	0.56	0.82
LOC100857837	1 ± 0.04	1.05 ± 0.09	1.24 ± 0.10	1.16 ± 0.12	1.01 ± 0.04	1.17 ± 0.21	0.41	0.71	0.67
*C2H8ORF22*	1 ± 0.13	1.20 ± 0.16	1.13 ± 0.11	1.12 ± 0.09	0.94 ± 0.08	0.79 ± 0.05	0.099	0.87	0.41
***SOX17***	1 ± 0.14	0.74 ± 0.07	0.83 ± 0.10	0.50 ± 0.08	0.32 ± 0.06	0.48 ± 0.04	0.00092	0.10	0.055
***LY96***	1 ± 0.12	0.75 ± 0.08	0.55 ± 0.07	0.42 ± 0.06	0.62 ± 0.12	0.92 ± 0.22	0.052	0.65	0.094
*CA2*	1 ± 0.47	0.71 ± 0.11	0.76 ± 0.23	1.97 ± 0.23	0.54 ± 0.11	0.61 ± 0.09	0.53	0.98	0.66
*ATP6V0D2*	1 ± 0.06	0.99 ± 0.12	1.11 ± 0.04	1.08 ± 0.10	0.98 ± 0.09	1.00 ± 0.09	0.47	0.94	0.97
*MMP16*	1 ± 0.02	0.57 ± 0.07	0.55 ± 0.06	0.33 ± 0.04	0.53 ± 0.10	0.33 ± 0.09	0.59	0.26	0.012
*NBN*	1 ± 0.05	1.07 ± 0.06	1.19 ± 0.08	1.21 ± 0.17	1.25 ± 0.12	1.57 ± 0.31	0.16	0.38	0.69
***CDH17***	1 ± 0.26	0.86 ± 0.17	0.78 ± 0.13	0.40 ± 0.07	0.38 ± 0.08	0.48 ± 0.09	0.020	0.31	0.38
***RNF151***	1 ± 0.23	1.19 ± 0.29	0.65 ± 0.07	0.68 ± 0.11	0.68 ± 0.02	0.54 ± 0.05	0.036	0.87	0.69
***PTDSS1***	1 ± 0.20	1.04 ± 0.24	1.44 ± 0.20	1.29 ± 0.16	0.70 ± 0.12	0.89 ± 0.21	0.060	0.87	0.75
*LAPTM4B*	1 ± 0.40	0.52 ± 0.05	0.48 ± 0.12	1.13 ± 0.31	0.42 ± 0.06	0.35 ± 0.09	0.20	0.88	0.10

Data are mean ± standard error for four individuals per sex per group; M, male; F, female. ^1^ ENSGALG# indicates the Ensembl Gene ID for *G*.* gallus*; official gene name not yet assigned. Genes in bold were selected for later analysis. ^2^ *p* values were obtained by two-way analysis of variance (ANOVA) to assess the effects of breed (B) and sex (S), as well as their interaction.

**Table 7:** Comparison of haplotype frequencies for nine genes between two extreme F_2_ groups with the highest and lowest PC1 scores.

Gene ^1^	Haplotype	Highest Group ^2^	Lowest Group ^2^	Chi-Square Value	*p* Value ^3^
*APCDD1*	NAG	0.42 (16)	0.38 (15)	0.17	0.68
	WPR	0.58 (22)	0.63 (25)		
ENSGALG00000051993	NAG	0.42 (16)	0.43 (17)	0.0010	0.97
	WPR	0.58 (22)	0.58 (23)		
ENSGALG00000053416	NAG	0.42 (15)	0.50 (18)	0.50	0.48
	WPR	0.58 (21)	0.50 (18)		
*NDC80*	NAG	0.42 (15)	0.50 (18)	0.50	0.48
	WPR	0.58 (21)	0.50 (18)		
*SOX17*	NAG	0.44 (15)	0.53 (18)	0.53	0.47
	WPR	0.56 (19)	0.47 (16)		
*LY96*	NAG	0.50 (16)	0.53 (18)	0.057	0.81
	WPR	0.50 (16)	0.47 (16)		
***CDH17***	NAG	0.69 (22)	0.50 (13)	3.06	0.080
	WPR	0.31 (11)	0.50 (13)		
***RNF151***	NAG	0.69 (22)	0.50 (13)	3.06	0.080
	WPR	0.31 (11)	0.50 (13)		
*PTDSS1*	NAG	0.66 (21)	0.53 (17)	0.83	0.36
	WPR	0.34 (17)	0.47 (15)		

^1^ ENSGALG# indicates the Ensembl Gene ID for *G*.* gallus*; official gene name not yet assigned. Genes in bold were used for later analysis. ^2^ Haplotype frequencies were inferred from three diplotypes at the five SNP marker loci used in the QTL analysis. The number of haplotypes is indicated in parentheses. ^3^ *p* values were obtained by Pearson’s chi-square test (df = 1).

## Data Availability

The RNA-seq data were deposited in the DDBJ Sequence Read Archive under accession numbers DRR540127-DRR540132 (aishikawa-0018_Run_0001-0006) for male chickens and DRR540133-DRR540138 (aishikawa-0019_Run_0001-0006) for female chickens. The data presented in this study are available in the Supplementary Materials.

## Supplementary Materials

The following supporting information can be downloaded at: https://www.mdpi.com/article/10.3390/genes17010062/s1, Figure S1: Score plots (left) and factor loading plots (right) for the first and second principal components (PC1 and PC2) obtained from principal component analysis (PCA) of four traits affected by a QTL on chromosome 2; Figure S2: Genetic linkage map of 313 SNP markers developed in the F_2_ cross population between NAG and WPR breeds of chickens; Figure S3: LOD score plots on chicken chromosome 2 for body weight, pectoralis minor muscle weight, pectoralis major muscle weight, total pectoralis muscle weight, and two types of PC1 scores summarizing variation in all four traits and in the three muscle weights; Figure S4: Correlation analysis between liver expression levels of the *CDH17* and *RNF151* genes and five traits: body weight, pectoralis minor muscle weight, pectoralis major muscle weight, total pectoralis muscle weight, and the first principal component (PC1) scores derived from the four muscle traits; Table S1: Raw data for body weight and pectoralis muscle weights for the NAG and WPR breeds, and their F_1_ and F_2_ chickens used in this study; Table S2: Environmental factors adjusted for raw trait data; Table S3: Summary of the genetic linkage map used in this study; Table S4: Genome-wide significant LOD thresholds used for single-QTL genome scans; Table S5: List of genes included in the top six enriched Gene Ontology (GO) biological processes terms; Table S6. Expression differences of two genes between two F2 groups in each sex with the highest and lowest PC1 scores.

## Abbreviations

The following abbreviations are used in this manuscript:

QTL	quantitative trait loci
GWASs	genome-wide association studies
RNA-seq	RNA-sequencing
RT-qPCR	reverse transcription quantitative PCR
NAG	Nagoya
JAS	Japanese Agricultural Standards
WPR	White Plymouth Rock
LOD	logarithms of the odds
PCA	principal component analysis
PC1	first principal component
SNP	single nucleotide polymorphism
RAD-seq	restriction site-associated DNA sequencing
DEGs	differentially expressed gene
GO	Gene Ontology
KEGG	Kyoto Encyclopedia of Genes and Genomes
*YWHAZ*	tyrosine 3-monooxygenase/tryptophan 5-monooxygenase activation protein zeta
*TBP*	TATA-box binding protein
*ACTB*	actin, beta
*RPL32*	ribosomal protein L32
*GAPDH*	glyceraldehyde-3-phosphate dehydrogenase
ANOVA	analysis of variance
HSD	Tukey’s honest significant difference
*IMPACT*	impact RWD domain protein
*GATA6*	GATA binding protein 6
*CDH17*	cadherin-17
*RNF151*	ring finger protein 151
*IGF-1*	insulin-like growth factor 1
*Mstn*	myostatin
FSH	follicle-stimulating hormone
*Fshb*	FSHβ subunit

## References

[1] Mackay T. (2001). The genetic architecture of quantitative traits. Annu. Rev. Genet..

[2] Keane T.M., Goodstadt L., Danecek P., White M.A., Wong K., Yalcin B., Heger A., Agam A., Slater G., Goodson M. (2011). Mouse genomic variation and its effect on phenotypes and gene regulation. Nature.

[3] Albert F.W., Kruglya L. (2015). The role of regulatory variation in complex traits and disease. Nat. Rev. Genet..

[4] Ishikawa A. (2017). A strategy for identifying quantitative trait genes using gene expression analysis and causal analysis. Genes.

[5] Visscher P.M., Wray N.R., Zhang Q., Sklar P., McCarthy M.I., Brown M.A., Yang J. (2017). 10 Years of GWAS discovery: Biology, function, and translation. Am. J. Hum. Genet..

[6] Al-Barghouthi B.M., Rosenow W.T., Du K.-P., Heo J., Maynard R., Mesner L., Calabrese G., Nakasone A., Senwar B., Gerstenfeld L. (2022). Transcriptome-wide association study and eQTL colocalization identify potentially causal genes responsible for human bone mineral density GWAS associations. eLife.

[7] Ghoreishifar M., Macleod I.M., Chamberlain A.J., Liu Z., Lopdell T.J., Littlejohn M.D., Xiang R., Pryce J.E., Goddard M.E. (2025). An integrative approach to prioritize candidate causal genes for complex traits in cattle. PLoS Genet..

[8] Ochiai T., Sakaguchi M., Kawakami S.I., Ishikawa A. (2023). Identification of candidate genes responsible for innate fear behavior in the chicken. G3.

[9] Imamura Y., Tsudzuki M., Roszkowski S. (2021). Japanese Chickens: The Living Art of the World.

[10] Tsudzuki M., Onitsuka S., Akiyama R., Iwamizu M., Goto N., Nishibori M., Takahashi H., Ishikawa A. (2007). Identification of quantitative trait loci affecting shank length, body weight and carcass weight from the Japanese cockfighting chicken breed, Oh-Shamo (Japanese Large Game). Cytogenet. Genome Res..

[11] Goto T., Ishikawa A., Nishibori M., Tsudzuki M. (2019). A longitudinal quantitative trait locus mapping of chicken growth traits. Mol. Genet. Genom..

[12] Essa B.H., Suzuki S., Nagano A.J., Elkholya S.Z., Ishikawa A. (2021). QTL analysis for early growth in an intercross between native Japanese Nagoya and White Plymouth Rock chicken breeds using RAD sequencing-based SNP markers. Anim. Genet..

[13] Yoshida M., Ishikawa A., Goto T., Goto N., Nishibori M., Tsudzuki M. (2013). QTL mapping for meat color traits using the F2 intercross between the Oh-Shamo (Japanese Large Game) and White Leghorn chickens. J. Poult. Sci..

[14] Ishikawa A., Essa B.H., Nasr S.M., Suzuki S. (2021). Mapping QTLs for breast muscle weight in an F_2_ intercross between native Japanese Nagoya and White Plymouth Rock chicken breeds. Life.

[15] Goto T., Ishikawa A., Onitsuka S., Goto N., Fujikawa Y., Umino T., Nishibori M., Tsudzuki M. (2011). Mapping quantitative trait loci for egg production traits in an F2 intercross of Oh-Shamo and White Leghorn chickens. Anim. Genet..

[16] Goto T., Ishikawa A., Yoshida M., Goto N., Umino T., Nishibori M., Tsudzuki M. (2014). Quantitative trait loci mapping for egg external traits in F2 chickens. J. Poult. Sci..

[17] Goto T., Ishikawa A., Goto N., Nishibori M., Umino T., Tsudzuki M. (2014). Mapping of main-effect and epistatic quantitative trait loci for internal egg traits in an F2 resource population of chickens. J. Poult. Sci..

[18] Ishikawa A., Sakaguchi M., Nagano A.J., Suzuki S. (2020). Genetic architecture of innate fear behavior in chickens. Behav. Genet..

[19] Velasco V.V., Ochiai T., Tsudzuki M., Goto N., Ishikawa A. (2024). Quantitative trait loci mapping of innate fear behavior in day-old F2 chickens of Japanese Oh-Shamo and White Leghorn breeds using restriction site-associated DNA sequencing. Poult. Sci..

[20] Ishikawa A., Eledel M., Essa B.H. (2019). Differences in growth and fat deposition between White Plymouth Rock and Nagoya breeds as a tool for QTL analysis. Damanhour J. Vet. Sci..

[21] Broman K.W., Sen S. (2009). A Guide to QTL Mapping with R/qtl.

[22] Bolger A.M., Lohse M., Usadel B. (2014). Trimmomatic: A flexible trimmer for Illumina sequence data. Bioinformatics.

[23] Kim D., Paggi J.M., Park C., Bennett C., Salzberg S.L. (2019). Graph-based genome alignment and genotyping with *HISAT2* and *HISAT*-genotype. Nat. Biotechnol..

[24] Love M.I., Huber W., Anders S. (2014). Moderated estimation of fold change and dispersion for RNA-seq data with DESeq2. Genome Biol..

[25] Zhou Y., Zhou B., Pache L., Chang M., Khodabakhshi A.H., Tanaseichuk O., Benner C., Chanda S.K. (2019). Metascape provides a biologist-oriented resource for the analysis of systems-level datasets. Nat. Commun..

[26] Bélteky J., Agnvall B., Johnsson M., Wright D., Jensen P. (2016). Domestication and tameness: Brain gene expression in red junglefowl selected for less fear of humans suggests effects on reproduction and immunology. R. Soc. Open Sci..

[27] Borowska D., Rothwell L., Bailey R.A., Watson K., Kaiser P. (2016). Identification of stable reference genes for quantitative PCR in cells derived from chicken lymphoid organs. Vet. Immunol. Immunopathol..

[28] Bagés S., Estany J., Tor M., Pena R.N. (2015). Investigating reference genes for quantitative real-time PCR analysis across four chicken tissues. Gene.

[29] Huppert S.S., Schwartz R.E. (2023). Multiple facets of cellular homeostasis and regeneration of the mammalian liver. Annu. Rev. Physiol..

[30] Wang H., Xu Y., Yu D., Li D., Liu X., Du W. (2015). Insulin-like growth factor-1 (*IGF-1*) promotes myoblast proliferation and skeletal muscle growth of embryonic chickens via the PI3K/Akt signaling pathway. Cell Biol. Int..

[31] Zhang D., Xu F., Liu Y. (2024). Research progress on regulating factors of muscle fiber heterogeneity in poultry: A review. Poult. Sci..

[32] Wang Z., Tian W., Wang D., Guo Y., Cheng Z., Zhang Y., Li X., Zhi Y., Li D., Li Z. (2023). Comparative analyses of dynamic transcriptome profiles highlight key response genes and dominant isoforms for muscle development and growth in chicken. Genet. Sel. Evol..

[33] Khullar S., Wang D. (2023). Predicting brain-regional gene regulatory networks from multi-omics for Alzheimer’s disease phenotypes and Covid-19 severity. Hum. Mol. Genet..

[34] Tegally H., Kensler K.H., Dilmohamud A., Ghoorah A.W., Rebbeck T.R., Baichoo S. (2020). Discovering novel driver mutations from pan-cancer analysis of mutational and gene expression profiles. PLoS ONE.

[35] Hwang S., Rhee S.Y., Marcotte E.M., Lee I. (2011). Systematic prediction of gene function in Arabidopsis thaliana using a probabilistic functional gene network. Nat. Protoc..

[36] Lin Z., Zhang C., Zhang M., Xu D., Fang Y., Zhou Z., Chen X., Qin N., Zhang X. (2014). Targeting Cadherin-17 inactivates Ras/Raf/MEK/ERK signaling and inhibits cell proliferation in gastric cancer. PLoS ONE.

[37] Long Z.W., Zhou M.L., Fu J.W., Chu X.O., Wang Y.N. (2015). Association between cadherin-17 expression and pathological characteristics of gastric cancer: A meta-analysis. World J. Gastroenterol..

[38] Qiu H., Zhang L., Ren C., Zheng Z., Wu W., Luo H., Zhou Z., Xu R. (2013). Targeting *CDH17* suppresses tumor progression in gastric cancer by downregulating Wnt/β-catenin signaling. PLoS ONE.

[39] Buqué X., Martínez M.J., Cano A., Miquilena-Colina M.E., García-Monzón C., Aspichueta P., Ochoa B. (2010). A subset of dysregulated metabolic and survival genes is associated with severity of hepatic steatosis in obese Zucker rats. J. Lipid Res..

[40] Ou X., Yang J., Zeng H., Shao L. (2025). Histone acetylation regulated by histone deacetylases during spermatogenesis. Andrology.

[41] Hou C., Yang W. (2013). New insights to the ubiquitin–proteasome pathway (UPP) mechanism during spermatogenesis. Mol. Biol. Rep..

[42] Pirbaluty A.M., Mehrban H., Kadkhodaei S., Ravash R., Oryan A., Ghaderi Z.M., Smith J. (2022). Network meta-analysis of chicken microarray data following avian influenza challenge–a comparison of highly and lowly pathogenic strains. Genes.

[43] Ongaro L., Zhou X., Wang Y., Schultz H., Zhou Z., Buddle E.R.S., Brûlé E., Lin Y.-F., Schang G., Hagg A. (2025). Muscle-derived myostatin is a major endocrine driver of follicle-stimulating hormone synthesis. Science.

[44] Makino K., Ishikawa A. (2018). Genetic identification of *Ly75* as a novel quantitative trait gene for resistance to obesity in mice. Sci. Rep..

[45] Chen P.B., Chen R., LaPierre N., Chen Z., Mefford J., Marcus E., Heffel M.G., Soto D.C., Ernst J., Luo C. (2024). Complementation testing identifies genes mediating effects at quantitative trait loci underlying fear-related behavior. Cell Genom..

