# Supplementary material for: New Candidate Genes for a Chicken Pectoralis Muscle Weight QTL Identified by a Hypothesis-Free Integrative Genetic Approach
Source: Genes (Basel). 2026 Jan 5;17(1):62. doi: 10.3390/genes17010062 (PMC12841298; doi:10.3390/genes17010062)
Supplement: Supplementary file 1 [file genes-17-00062-s001.zip › Supplementary Figures.pdf]

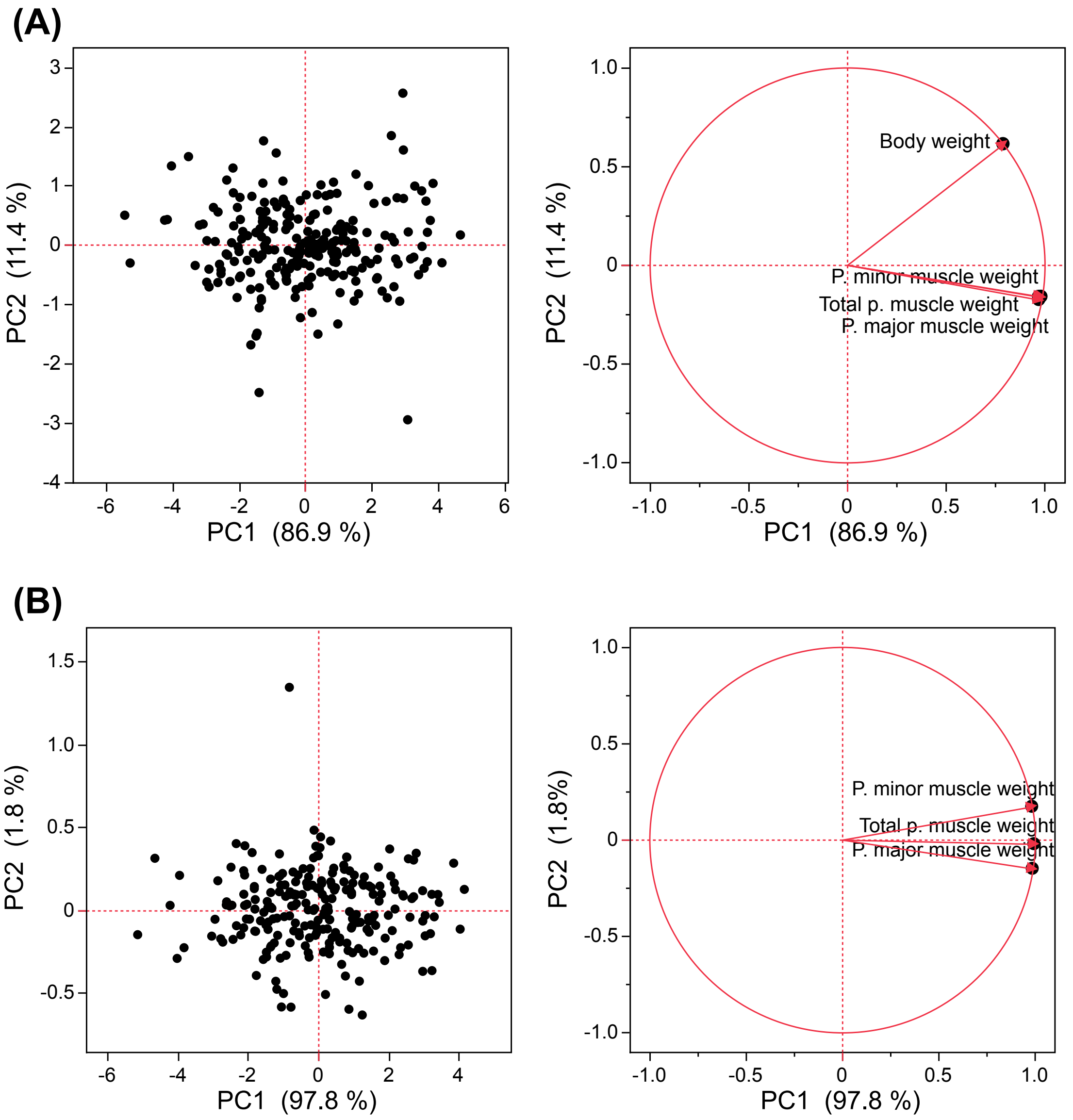

Supplementary Figure S1. Score plots (left) and factor loading plots (right) for the first and second principal components (PC1 and PC2) obtained from principal component analysis (PCA) of four traits affected by a QTL on chromosome 2. (A) PCA of body weight and the weights of three pectoralis muscles; (B) PCA of the three pectoralis muscle weights only. The percentage of total variance explained by each axis is shown in parentheses.

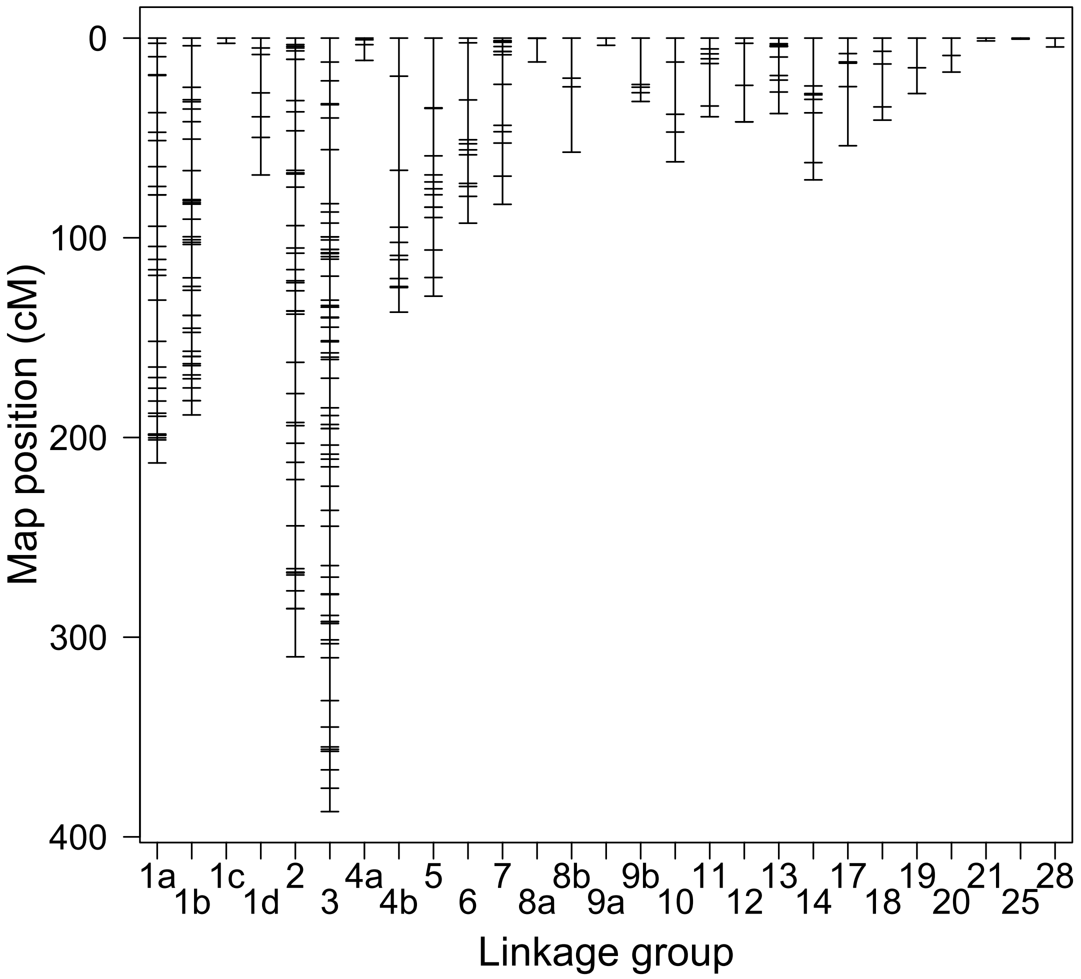

Supplementary Figure S2. Genetic linkage map of 313 SNP markers developed in the F_2 cross population between NAG and WPR breeds of chickens. Horizontal lines indicate the map positions of the SNP markers. Numbers in linkage groups indicate chromosome numbers, and letters show specific linkage groups within chromosomes 1, 4, 8, and 9. See Supplementary Table S3 for detailed parameters of the linkage groups.

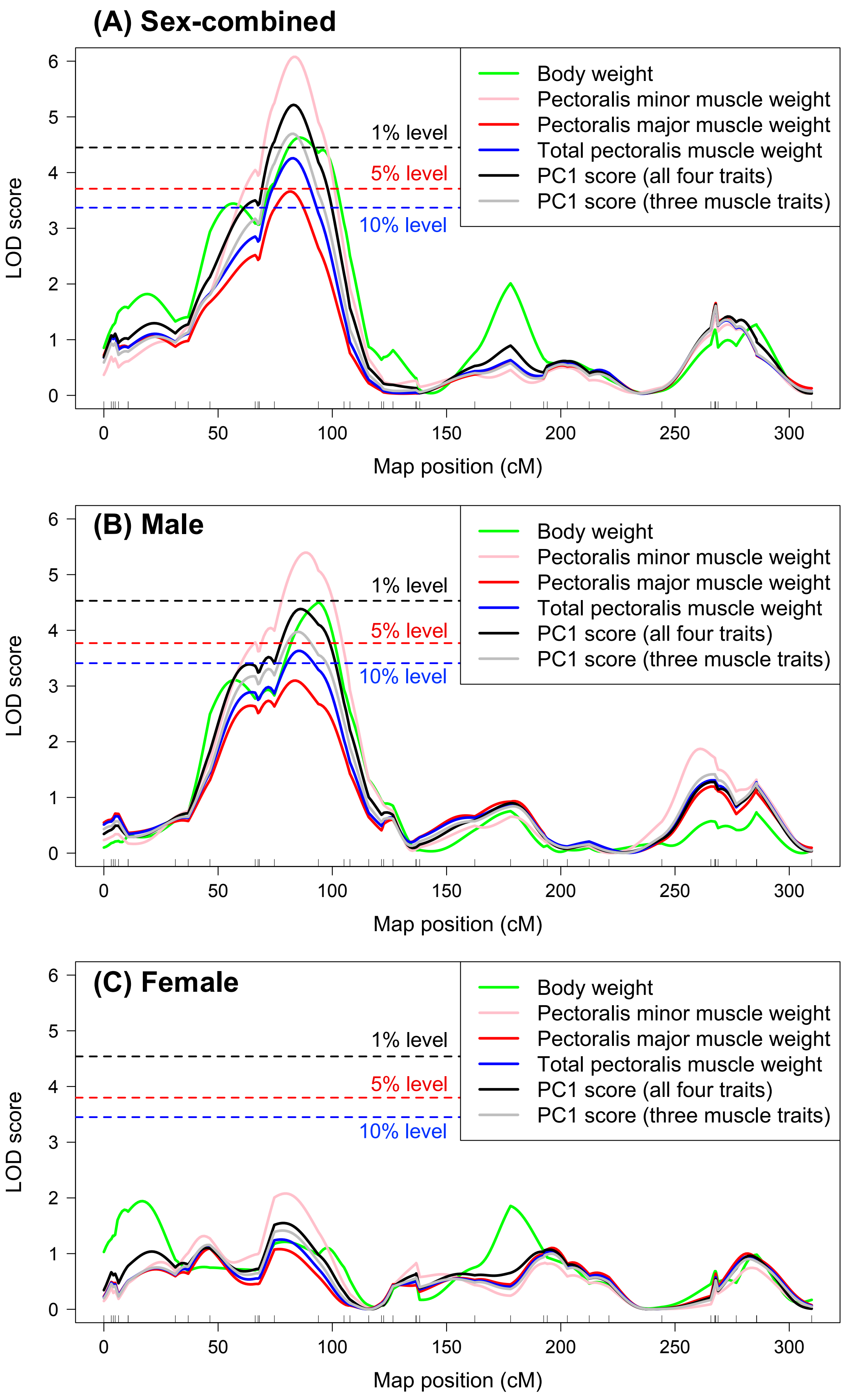

Supplementary Figure S3. (continued)

Supplementary Figure S3. LOD score plots on chicken chromosome 2 for body weight, pectoralis minor muscle weight, pectoralis major muscle weight, total pectoralis muscle weight, and two types of PC1 scores summarizing variation in all four traits and in the three muscle weights. (A) Sex-combined data, (B) male data, and (C) female data. Horizontal dashed lines indicate significance thresholds determined by 10,000 permutation tests (see Supplementary Table S4 for threshold values).

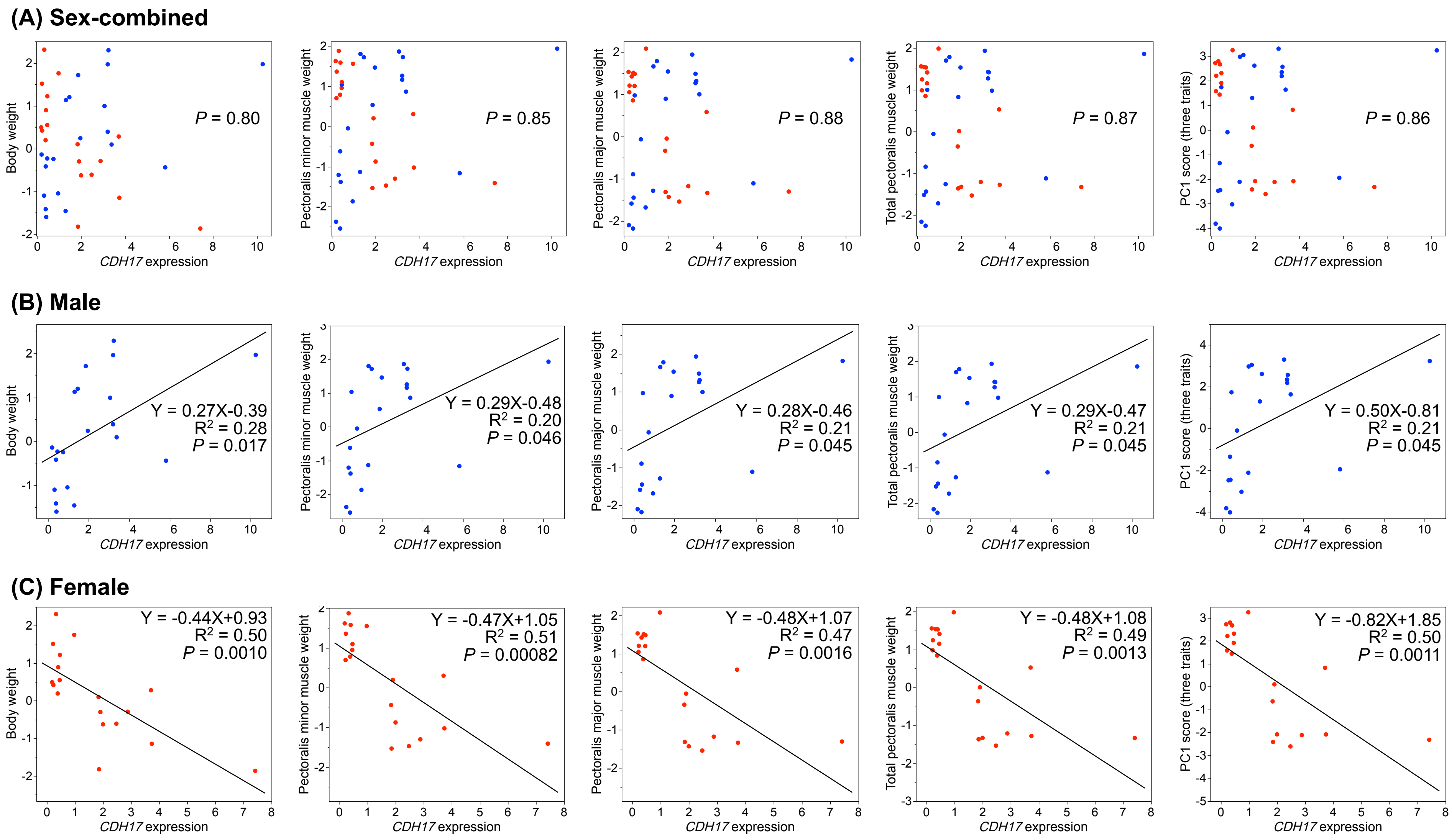

Supplementary Figure S4. (continued)

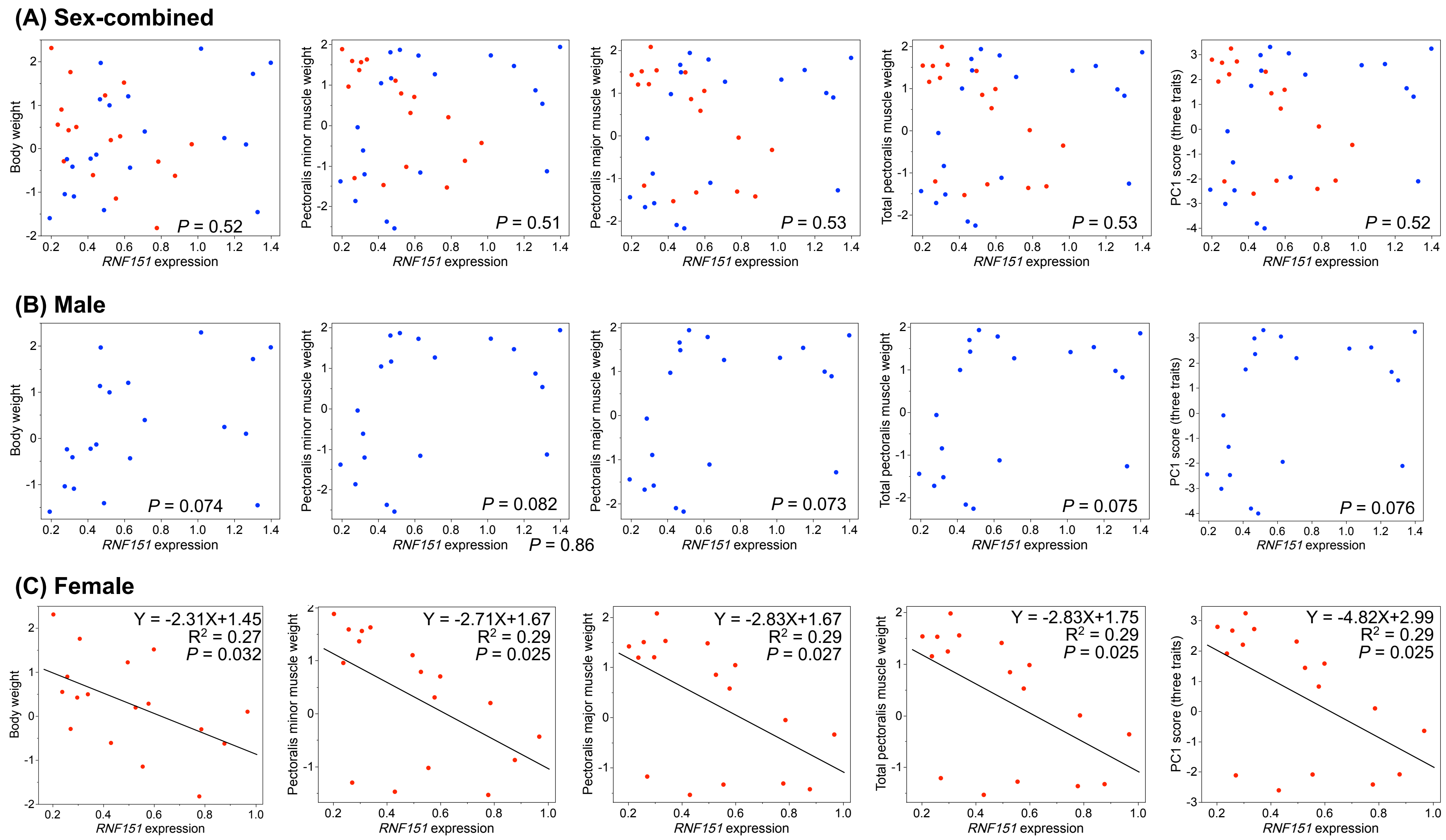

Supplementary Figure S4. (continued)

Supplementary Figure S4. Correlation analysis between liver expression levels of the *CDH17* and *RNF151* genes and five traits: body weight, pectoralis minor muscle weight, pectoralis major muscle weight, total pectoralis muscle weight, and the first principal component (PC1) scores derived from the four muscle traits. Analyses were performed by sex using F_2 individuals with the highest (n = 10) and lowest (n = 9) PC1 scores in each sex. Closed blue and red circles indicate males and females, respectively. (A) sex-combined data, (B) male data only, and (C) female data only.
